# Adverse event signals associated with targeted therapy plus chemotherapy in colorectal cancer: A FAERS-based pharmacovigilance study

**DOI:** 10.1097/MD.0000000000051207

**Published:** 2026-11-06

**Authors:** Youqiang Liu, Zhihan Li, Gang Li, Hengxue Lin

**Affiliations:** a The Second Department of General Surgery, The Fourth Hospital of Hebei Medical University, Shijiazhuang, China; b Department of Gastrointestinal Surgery, Affiliated Hospital of Hebei University, Baoding, Hebei, China.

**Keywords:** bevacizumab, cetuximab, colorectal cancer, pharmacovigilance, thrombocytopenia

## Abstract

Real-world safety profiles of targeted therapies for colorectal cancer (CRC) remain incompletely characterized, particularly across different chemotherapy backbones. This study characterizes adverse event (AE) reporting patterns for bevacizumab (BVZ), cetuximab (C225), and panitumumab (VEC), used as monotherapy or in combination with folinic acid, fluorouracil, and oxaliplatin (FOLFOX) or folinic acid, fluorouracil, and irinotecan (FOLFIRI), within CRC-related reports. CRC-related AE reports from 2006 to 2025 for BVZ, C225, and VEC were extracted from the U.S. FDA Adverse Event Reporting System. Disproportionality signals were evaluated using 4 algorithms: the reporting odds ratio (ROR), proportional reporting ratio, Bayesian confidence propagation neural network, and multi-item gamma Poisson shrinker. Time-to-onset, regimen-stratified, and exploratory univariable logistic regression analyses were also conducted. The non-case reference population was restricted to the CRC-related reporting subcohort rather than the entire FDA Adverse Event Reporting System database. A total of 38,117 reports were identified, mainly involving BVZ (n = 20,521). Hematologic, hepatobiliary, and gastrointestinal toxicities showed prominent reporting signals across regimens (ROR = 4.15, 2.84, and 2.46, respectively). BVZ was associated with proteinuria and hand-foot syndrome, while C225 and VEC showed prominent cutaneous and marrow-suppression signals. Nine AE terms overlapped across agents, including thrombocytopenia, with fatality proportions of 23%, 13%, and 10% for BVZ, C225, and VEC, respectively. Median onset of nonfatal thrombocytopenia differed by agent: 121 days for BVZ, 6 days for C225, and 40 days for VEC; fatal C225-associated events also occurred early (median 6 days, *P *= .006). FOLFOX strengthened thrombocytopenia signals, especially with VEC-FOLFOX (ROR = 10.5) and C225-FOLFOX (ROR = 9.3), and peripheral neuropathy was reported across FOLFOX-containing regimens. CRC targeted therapies showed agent- and regimen-specific AE reporting patterns. Thrombocytopenia varied by onset and fatality and was more prominent with FOLFOX-containing regimens. These findings support tailored monitoring, particularly platelet surveillance for C225 and FOLFOX-based combinations, with longer-term vigilance for BVZ. Given the limitations of spontaneous reporting, including potential co-medication, indication-related, and residual confounding, these signals should not be interpreted as establishing causality.

## 1. Introduction

Colorectal cancer (CRC) remains a major global health burden, with an estimated ~1.9 million new cases and ~0.9 million deaths (DEs) worldwide in 2022.^[1]^ In the U.S., CRC continues to contribute substantially to cancer incidence and mortality, reinforcing the need to optimize both efficacy and safety of systemic therapies used across the disease course.^[2]^ For patients with unresectable or metastatic CRC, contemporary treatment is anchored by fluoropyrimidine-based combination chemotherapy (typically folinic acid, fluorouracil, and oxaliplatin [FOLFOX] or folinic acid, fluorouracil, and irinotecan [FOLFIRI]) and the integration of targeted biologics selected by clinicopathologic factors and tumor biomarkers.^[3,4]^ In particular, anti-vascular endothelial growth factor (VEGF) therapy with bevacizumab (BVZ) and anti-epidermal growth factor receptor (EGFR) monoclonal antibodies (cetuximab [C225] and panitumumab [VEC]) are foundational options used as monotherapy in selected settings and, more commonly, in combination with chemotherapy backbones. The clinical value of these agents is supported by pivotal randomized trials demonstrating improved outcomes when biologics are added to standard chemotherapy regimens in molecularly appropriate populations.^[5–7]^

However, treatment optimization in CRC is increasingly constrained not only by efficacy trade-offs but also by regimen-specific toxicity. Anti-VEGF therapy is associated with characteristic vascular–renal and bleeding-related risks (e.g., hypertension, hemorrhage, gastrointestinal perforation, and proteinuria), while anti-EGFR therapy commonly induces dermatologic and nail toxicities and can be associated with electrolyte abnormalities and uncommon but serious pulmonary events such as interstitial lung disease.^[8–11]^ In parallel, chemotherapy backbones contribute their own toxicity signatures, most notably oxaliplatin-associated peripheral neuropathy in FOLFOX-based regimens.^[12]^ Hematologic toxicities, including thrombocytopenia, remain clinically consequential because they can precipitate bleeding, trigger dose delays or reductions, and compromise treatment intensity – an issue increasingly emphasized in modern supportive care guidance.^[12,13]^

Despite extensive trial and guideline evidence, important gaps remain. First, randomized trials and conventional safety summaries are often underpowered to characterize rare, serious, or delayed adverse events (AEs), and they may not capture how toxicity patterns shift when targeted agents are paired with specific chemotherapy backbones in real-world practice.^[3,4]^ Second, comparative, regimen-stratified safety evidence across commonly used targeted strategies (BVZ-, C225-, and VEC-based approaches) is limited, particularly for outcomes where clinical risk is shaped by both the biologic agent and chemotherapy context (e.g., thrombocytopenia, pulmonary toxicity, and neuropathy). Third, beyond “what” AEs occur, clinicians frequently need actionable information on severity (including fatality), time-to-onset (TTO), and patient/report-level correlates to guide monitoring intensity, early recognition, and supportive interventions – dimensions that are not consistently detailed in routine trial reporting and are difficult to reconstruct from heterogeneous real-world datasets.

Spontaneous reporting systems offer a complementary approach to address these gaps. The U.S. FDA Adverse Event Reporting System (FAERS)^[14]^ aggregates large-scale postmarketing individual case safety reports across broad populations and long-time horizons, enabling detection of uncommon events and evaluation of real-world toxicity patterns not readily observable in trials. At the same time, FAERS requires careful interpretation because reports are subject to underreporting, stimulated reporting, missingness, duplicates/follow-ups, and the fundamental limitation that reporting does not establish causality.^[15]^ Accordingly, pharmacovigilance studies commonly use disproportionality methods (e.g., reporting odds ratio [ROR]) within a case–noncase framework to screen for signals while emphasizing transparent reporting and cautious inference.^[16–19]^ Standardized event coding using Medical Dictionary for Regulatory Activities further supports reproducible analyses at both broad System Organ Class (SOC) and granular preferred term (PT) levels.^[20,21]^

The current study aims to leverage FAERS to systematically characterize AE patterns associated with major targeted therapies used in CRC with a focus on regimen-stratified comparisons. By mapping toxicity domains across agents and chemotherapy backbones in a large postmarketing dataset, this work aims to fill a practical evidence gap, providing regimen-tailored pharmacovigilance insights that can inform monitoring priorities, supportive care planning, and hypothesis generation for prospective validation.

## 2. Methods

### 2.1. Data source and AE identification

This study utilized publicly available, de-identified data from the FAERS. As no direct human subjects were involved, ethical approval and informed consent were not required. Individual case safety reports were retrieved from the U.S. FAERS through the FDA website. We downloaded the quarterly ASCII datasets from Q1 2006 to Q3 2025 and harmonized variables across reporting periods. To maximize the capture of relevant reports, drug names were mapped using synonyms and aliases curated from the NIH Medical Subject Headings database. AEs were mapped to the most recent version of Medical Dictionary for Regulatory Activities, and corresponding SOCs and PTs were used for downstream analyses.

### 2.2. Data preprocessing, eligibility criteria, and missing data

FAERS data were cleaned in accordance with FDA-recommended procedures. Duplicates were first addressed at the case level using the DEMO table (PRIMARYID, CASEID, and FDA_DT). For reports sharing the same CASEID, records were ordered by FDA_DT and retained based on the most recent FDA_DT; if FDA_DT was identical, the record with the largest PRIMARYID was kept. We then performed additional deduplication by removing records with identical values across key fields (e.g., sex, age, country, event date, reported PTs, drug entries, and indications).

Eligible reports were those involving patients with CRC in which a targeted therapy, including BVZ, C225, or VEC, was recorded as the primary suspect (PS) drug and was used alone or in combination with chemotherapy agents, including 5-fluorouracil, oxaliplatin, irinotecan, or capecitabine. Reports were excluded if the targeted agent was recorded only as a concomitant medication, if the indication was not CRC, if key drug or AE coding information was missing, or if the AE onset date clearly preceded the drug start date. Duplicate reports were removed as described above.

For descriptive analyses, missing values were retained as a separate “Missing” category, and the proportion of missing data was reported for relevant variables. For stratified analyses, complete-case analysis was performed by excluding reports with missing values for key covariates, including age, sex, and body weight. We did not conduct multiple imputations to avoid introducing additional assumptions or potential bias.

### 2.3. Disproportionality signal detection

FAERS assigns a drug role code (ROLE_COD) in the DRUG file to indicate the reported causal attribution: PS, secondary suspect, concomitant (C), or interacting (I). To improve specificity for drug–event attribution, analyses were restricted to reports in which the drug of interest was coded as PS. Because FAERS is a spontaneous reporting system, it does not provide information on the total number of patients exposed to each drug or the incidence rates of AEs. Therefore, FAERS-based disproportionality analyses cannot estimate absolute or relative risk and should be interpreted as signal-detection analyses rather than causal risk assessments. Drug–AE associations were assessed using standard disproportionality approaches based on a 2 × 2 contingency table, including the ROR, proportional reporting ratio (PRR), Bayesian confidence propagation neural network (BCPNN), and multi-item gamma Poisson shrinker (MGPS). In the contingency table, *a* represents the number of reports containing both the target drug and target AE, *b* represents reports containing the target drug and other AEs, *c* represents reports containing the target AE and other eligible drugs, and *d* represents reports containing other eligible drugs and other AEs. The comparator population comprised all other eligible reports within the CRC-restricted FAERS subcohort rather than the entire FAERS database. These methods were used because FAERS data are subject to underreporting, incomplete reporting, duplicate reports, and reporting bias, and no single algorithm provides an optimal balance between sensitivity and specificity. ROR and PRR are frequency-based methods that are straightforward to calculate, easy to interpret, and sensitive for initial signal detection. In contrast, BCPNN and MGPS are Bayesian shrinkage methods that provide more conservative estimates and are useful for reducing instability caused by sparse reporting. Therefore, we applied multiple complementary algorithms to improve the robustness of signal detection, while using ROR as the primary metric because of its interpretability and widespread use in pharmacovigilance studies. For ROR-based signal detection, a signal was defined as at least 3 reports and a lower bound of the 95% confidence interval (CI) of at least 1.^[22]^

The calculation of ROR is based on the formulas below:


$$ROR=\frac{ad}{bc}$$

$$\begin{array}{c} SE\left(lnROR\right)=\sqrt{\left(\frac{1}{a}+\frac{1}{b}+\frac{1}{c}+\frac{1}{d}\right)} \end{array}$$

$$\begin{array}{c} 95\%CI=e^{ln\left(ROR\right)\pm \sqrt[{1.96}]{\left(\frac{1}{a}+\frac{1}{b}+\frac{1}{c}+\frac{1}{d}\right)}} \end{array}$$

where, *a* denotes the number of reports listing both the target drug and target event; *b*, the target drug with other events; *c*, the target event with other drugs; and *d*, other drugs with other events. For PRR, a signal required *a* ≥ 3, PRR ≥ 2, and a lower bound of the 95% CI ≥ 1. For BCPNN, signal strength was expressed using the information component (IC), and a positive signal was defined as IC_025_ > 0, where IC_025_ represents the lower bound of the 95% credibility interval and was estimated as \(E(IC)-2 standard deviation [SD]\). For MGPS, signal strength was expressed using the empirical Bayes geometric mean (EBGM), and a positive signal was defined as EBGM_05_ ≥ 2, where EBGM_05_ represents the lower 5th percentile of the posterior distribution. These methods and signal-detection thresholds were applied in accordance with previous methodology publication.^[22]^

### 2.4. TTO analysis

TTO was defined as the interval between treatment initiation and AE occurrence. Distributions were summarized using the median and interquartile range (IQR) and modeled using a Weibull distribution. The Weibull scale (α) and shape (β) parameters were estimated to characterize time-to-event patterns, where β < 1 suggests a decreasing hazard over time (early-failure pattern), β ≈ 1 indicates an approximately constant hazard (random-failure pattern), and β > 1 reflects an increasing hazard over time (wear-out pattern).

### 2.5. Statistical analysis

To further identify the key populations experiencing AEs, we conducted stratified analyses based on clinical factors, including sex, age, and calendar quarter. Herein, age was categorized as < 18 years, 18–65 years, and ≥ 65 years. Statistical significance was assessed using a two-sided threshold of *P* < .05.

All analyses were conducted in R (v4.5.1). Continuous variables (e.g., age and body weight) were summarized as mean (SD), and categorical variables (e.g., sex, age/weight strata, reporter type, and outcomes) as counts and percentages. Two-group comparisons used independent-samples *t* tests; comparisons across multiple groups used one-way ANOVA. Outcomes were further classified by severity into severe (DE, disability, or life-threatening [LT]) and moderate (hospitalization [HO] or other serious outcomes [OTs]) categories, and between-group differences were evaluated using chi-square tests. A two-sided *P* < .05 was considered statistically significant.

## 3. Results

### 3.1. Baseline characteristics of AEs and patient population

Following data processing, a total of 38,117 AE reports related to targeted therapies for CRC were identified in the FAERS database between 2006 and 2025. Most reports involved BVZ, including BVZ monotherapy (n = 14,755), BVZ + FOLFIRI (n = 2460), and BVZ + FOLFOX (n = 3306). For C225, counts were 6095 for monotherapy, 728 for C225 + FOLFIRI, and 820 for C225 + FOLFOX. For VEC, we identified 6991 reports for monotherapy, along with 1157 for VEC + FOLFIRI and 1805 for VEC + FOLFOX (Fig. 1). Reporting volume increased substantially over time, although year-to-year fluctuations were evident. The lowest annual total occurred in 2010 (n = 825), whereas reporting peaked in 2017 (n = 2531). Reporting was relatively low and stable before 2010, then rose sharply after 2012 and stayed elevated through the remainder of the period. Even after a modest decline from the 2017 peak, yearly totals remained consistently high, with more than 1800 reports per year thereafter.

**Figure 1.**
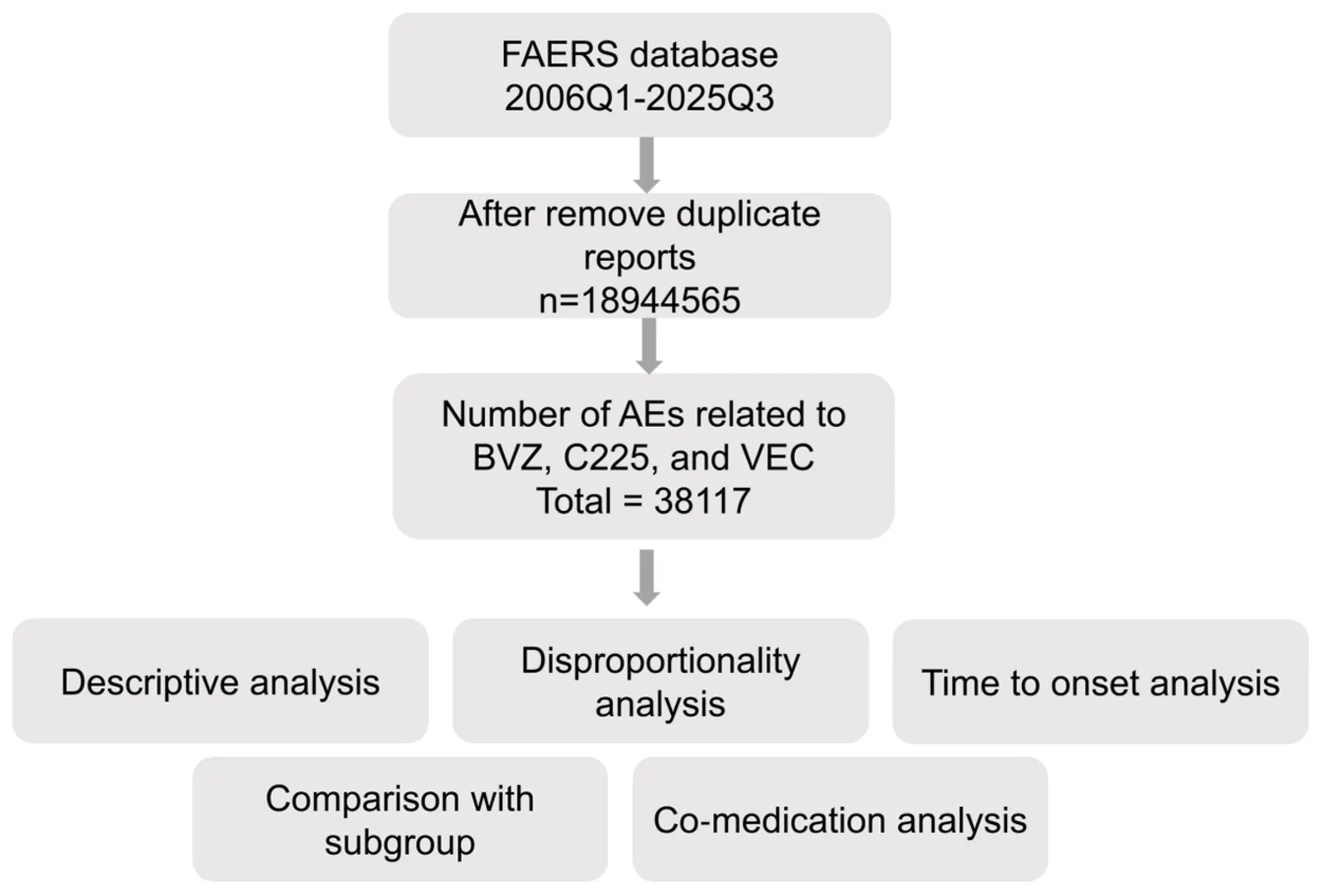
Schematic overview of the analytical framework. AE = adverse event, BVZ = bevacizumab, C225 = cetuximab, FAERS = FDA Adverse Event Reporting System, VEC = panitumumab.

In terms of the distribution of AE reports by sex, we found that the majority of reports involved male patients (n = 20,009; 52.5% vs 33.2% and n = 12,643 in females), while sex was unknown in 5465 cases (14.3%). The number of AE reports among men was approximately 1.6 times higher than among women. Adults represented the largest age group (n = 14,892; 39.1%), followed by older adults (n = 12,575; 33.0%), whereas adolescents were rarely represented (n = 31; 0.1%). The mean patient age was 63.36 (SD = 12.96) years. Among patients with available weight data, the majority fell within the 50 to 100 kg range (n = 10,318; 27.1%), followed by those weighing < 50 kg (n = 1930; 5.1%). Individuals weighing > 100 kg were least represented (n = 631; 1.7%). The mean body weight was 68.07 (SD = 18.77) kg.

Most AE reports were submitted by healthcare professionals (n = 28,677; 75.2%), whereas non-healthcare reporters, including patients and family members, accounted for 9122 reports (23.9%); reporter identity was unknown in 318 cases (0.8%). The predominance of professionally submitted reports supports the overall clinical credibility of the dataset. Among reports with documented outcomes, “other serious illness” was the most frequently recorded outcome (n = 21,425; 44.2%), followed by HO (n = 13,202; 27.2%), DE (n = 7915; 16.3%), LT events (n = 2525; 5.2%), and disability (n = 712; 1.5%). These findings suggest that while AEs associated with targeted therapies in CRC are predominantly serious, the proportion resulting in permanent disability remains relatively low (Table 1; Fig. 2).

**Table 1:** Demographic characteristics of patients with adverse events.

	Level	Overall	BVZ	BVZ_FOLFIRI	BVZ_FOLFOX	C225	C225_FOLFIRI	C225_FOLFOX	VEC	VEC_FOLFIRI	VEC_FOLFOX	*P*
n		38,117	14,755	2460	3306	6095	728	820	6991	1157	1805	
Year (%)	2006	1057 (3.0)	514 (3.7)	57 (2.4)	140 (4.4)	292 (5.4)	8 (1.2)	16 (2.0)	19 (0.3)	4 (0.4)	7 (0.4)	<.001
	2007	1062 (3.0)	483 (3.5)	39 (1.6)	99 (3.1)	213 (3.9)	5 (0.7)	10 (1.2)	151 (2.4)	26 (2.4)	36 (2.1)	
	2008	1409 (4.0)	696 (5.0)	63 (2.6)	147 (4.6)	210 (3.9)	10 (1.5)	32 (4.0)	172 (2.8)	47 (4.3)	32 (1.8)	
	2009	1212 (3.4)	602 (4.4)	45 (1.9)	53 (1.7)	251 (4.6)	14 (2.0)	6 (0.7)	196 (3.1)	28 (2.5)	17 (1.0)	
	2010	825 (2.3)	348 (2.5)	18 (0.8)	19 (0.6)	244 (4.5)	15 (2.2)	8 (1.0)	161 (2.6)	7 (0.6)	5 (0.3)	
	2011	1109 (3.1)	325 (2.3)	54 (2.3)	65 (2.0)	152 (2.8)	20 (2.9)	14 (1.7)	373 (6.0)	60 (5.4)	46 (2.7)	
	2012	1943 (5.5)	1002 (7.2)	116 (4.9)	170 (5.3)	241 (4.5)	26 (3.8)	26 (3.2)	273 (4.4)	41 (3.7)	48 (2.8)	
	2013	1709 (4.8)	1120 (8.1)	42 (1.8)	76 (2.4)	234 (4.3)	6 (0.9)	5 (0.6)	211 (3.4)	7 (0.6)	8 (0.5)	
	2014	1779 (5.0)	848 (6.1)	121 (5.1)	134 (4.2)	241 (4.5)	37 (5.4)	21 (2.6)	295 (4.7)	30 (2.7)	52 (3.0)	
	2015	2119 (6.0)	817 (5.9)	204 (8.6)	284 (8.9)	267 (4.9)	49 (7.2)	28 (3.5)	323 (5.2)	65 (5.9)	82 (4.7)	
	2016	1993 (5.6)	680 (4.9)	113 (4.7)	162 (5.1)	345 (6.4)	69 (10.1)	44 (5.5)	393 (6.3)	59 (5.3)	128 (7.4)	
	2017	2531 (7.2)	753 (5.4)	167 (7.0)	238 (7.4)	455 (8.4)	74 (10.8)	133 (16.5)	496 (8.0)	67 (6.1)	148 (8.5)	
	2018	2053 (5.8)	766 (5.5)	179 (7.5)	207 (6.5)	229 (4.2)	41 (6.0)	44 (5.5)	393 (6.3)	74 (6.7)	120 (6.9)	
	2019	2237 (6.3)	720 (5.2)	171 (7.2)	241 (7.5)	240 (4.4)	45 (6.6)	38 (4.7)	490 (7.9)	116 (10.5)	176 (10.2)	
	2020	2195 (6.2)	754 (5.4)	217 (9.1)	254 (7.9)	219 (4.1)	36 (5.3)	54 (6.7)	419 (6.7)	71 (6.4)	171 (9.9)	
	2021	1766 (5.0)	601 (4.3)	123 (5.2)	149 (4.7)	244 (4.5)	30 (4.4)	46 (5.7)	375 (6.0)	74 (6.7)	124 (7.2)	
	2022	2166 (6.1)	790 (5.7)	161 (6.8)	199 (6.2)	269 (5.0)	50 (7.3)	69 (8.6)	372 (6.0)	99 (9.0)	157 (9.1)	
	2023	2384 (6.7)	791 (5.7)	202 (8.5)	241 (7.5)	356 (6.6)	63 (9.2)	67 (8.3)	398 (6.4)	109 (9.9)	157 (9.1)	
	2024	2021 (5.7)	612 (4.4)	131 (5.5)	166 (5.2)	371 (6.9)	48 (7.0)	76 (9.5)	401 (6.4)	79 (7.1)	137 (7.9)	
	2025	1801 (5.1)	613 (4.4)	162 (6.8)	152 (4.8)	330 (6.1)	38 (5.6)	67 (8.3)	317 (5.1)	42 (3.8)	80 (4.6)	
Sex (%)	Female	12,643 (33.2)	5075 (34.4)	756 (30.7)	1074 (32.5)	2024 (33.2)	229 (31.5)	285 (34.8)	2284 (32.7)	337 (29.1)	579 (32.1)	<.001
	Male	20,009 (52.5)	7332 (49.7)	1163 (47.3)	1588 (48.0)	3454 (56.7)	452 (62.1)	407 (49.6)	3870 (55.4)	683 (59.0)	1060 (58.7)	
	Unknown	5465 (14.3)	2348 (15.9)	541 (22.0)	644 (19.5)	617 (10.1)	47 (6.5)	128 (15.6)	837 (12.0)	137 (11.8)	166 (9.2)	
Age Group (%)	Juvenile	31 (0.1)	18 (0.1)	1 (0.0)	3 (0.1)	2 (0.0)	0 (0.0)	1 (0.1)	4 (0.1)	1 (0.1)	1 (0.1)	<.001
	Adult	14,892 (39.1)	5334 (36.2)	1010 (41.1)	1321 (40.0)	2728 (44.8)	349 (47.9)	391 (47.7)	2605 (37.3)	437 (37.8)	717 (39.7)	
	Seniors	12,575 (33.0)	4342 (29.4)	709 (28.8)	1025 (31.0)	2158 (35.4)	275 (37.8)	261 (31.8)	2596 (37.1)	451 (39.0)	758 (42.0)	
	Unknown	10,619 (27.9)	5061 (34.3)	740 (30.1)	957 (28.9)	1207 (19.8)	104 (14.3)	167 (20.4)	1786 (25.5)	268 (23.2)	329 (18.2)	
Age (mean [SD])		63.36 (12.96)	62.97 (12.81)	61.88 (12.62)	62.60 (13.05)	62.61 (11.75)	62.08 (11.85)	60.76 (12.07)	65.08 (14.08)	65.56 (13.07)	65.73 (13.49)	<.001
Weight group (%)	≤50kg	1930 (5.1)	750 (5.1)	109 (4.4)	147 (4.4)	316 (5.2)	39 (5.4)	56 (6.8)	351 (5.0)	60 (5.2)	102 (5.7)	<.001
	50–100kg	10,318 (27.1)	4067 (27.6)	642 (26.1)	879 (26.6)	1764 (28.9)	257 (35.3)	219 (26.7)	1679 (24.0)	326 (28.2)	485 (26.9)	
	>100kg	631 (1.7)	201 (1.4)	39 (1.6)	56 (1.7)	143 (2.3)	12 (1.6)	12 (1.5)	121 (1.7)	23 (2.0)	24 (1.3)	
	Unknown	25,238 (66.2)	9737 (66.0)	1670 (67.9)	2224 (67.3)	3872 (63.5)	420 (57.7)	533 (65.0)	4840 (69.2)	748 (64.6)	1194 (66.1)	
Weight (mean [SD])		68.07 (18.77)	67.75 (18.06)	70.07 (18.40)	69.30 (19.22)	69.24 (20.07)	68.51 (19.05)	64.22 (17.15)	67.63 (19.46)	67.81 (18.60)	64.96 (16.76)	<.001
Reporter (%)	Healthcare professional	28,677 (75.2)	11,797 (80.0)	2277 (92.6)	3061 (92.6)	2064 (33.9)	201 (27.6)	266 (32.4)	6115 (87.5)	1140 (98.5)	1756 (97.3)	<.001
	Non-healthcare professional	9122 (23.9)	2746 (18.6)	150 (6.1)	200 (6.0)	4019 (65.9)	526 (72.3)	553 (67.4)	863 (12.3)	17 (1.5)	48 (2.7)	
	Unknown	318 (0.8)	212 (1.4)	33 (1.3)	45 (1.4)	12 (0.2)	1 (0.1)	1 (0.1)	13 (0.2)	0 (0.0)	1 (0.1)	
Outcome (%)	Death	7915 (16.3)	3951 (22.0)	567 (18.3)	711 (17.0)	635 (8.6)	71 (7.9)	103 (10.2)	1368 (13.9)	216 (13.1)	293 (11.6)	<.001
	Disability	712 (1.5)	214 (1.2)	52 (1.7)	34 (0.8)	163 (2.2)	20 (2.2)	32 (3.2)	155 (1.6)	16 (1.0)	26 (1.0)	
	Hospitalization	13,202 (27.2)	4827 (26.9)	737 (23.8)	1145 (27.3)	2285 (31.0)	282 (31.5)	296 (29.2)	2553 (26.0)	447 (27.1)	630 (25.0)	
	Life-threatening	2525 (5.2)	832 (4.6)	117 (3.8)	209 (5.0)	528 (7.2)	60 (6.7)	54 (5.3)	507 (5.2)	93 (5.6)	125 (5.0)	
	Other serious illness	21,425 (44.2)	7211 (40.2)	1548 (50.0)	1980 (47.3)	3076 (41.7)	419 (46.8)	511 (50.5)	4484 (45.6)	824 (50.0)	1372 (54.4)	
	Unknown	2746 (5.7)	920 (5.1)	75 (2.4)	110 (2.6)	692 (9.4)	44 (4.9)	16 (1.6)	763 (7.8)	52 (3.2)	74 (2.9)	

BVZ = bevacizumab, C225 = cetuximab, FOLFIRI = folinic acid, fluorouracil, and Irinotecan, FOLFOX = folinic acid, fluorouracil, and oxaliplatin, VEC = panitumumab.

**Figure 2.**
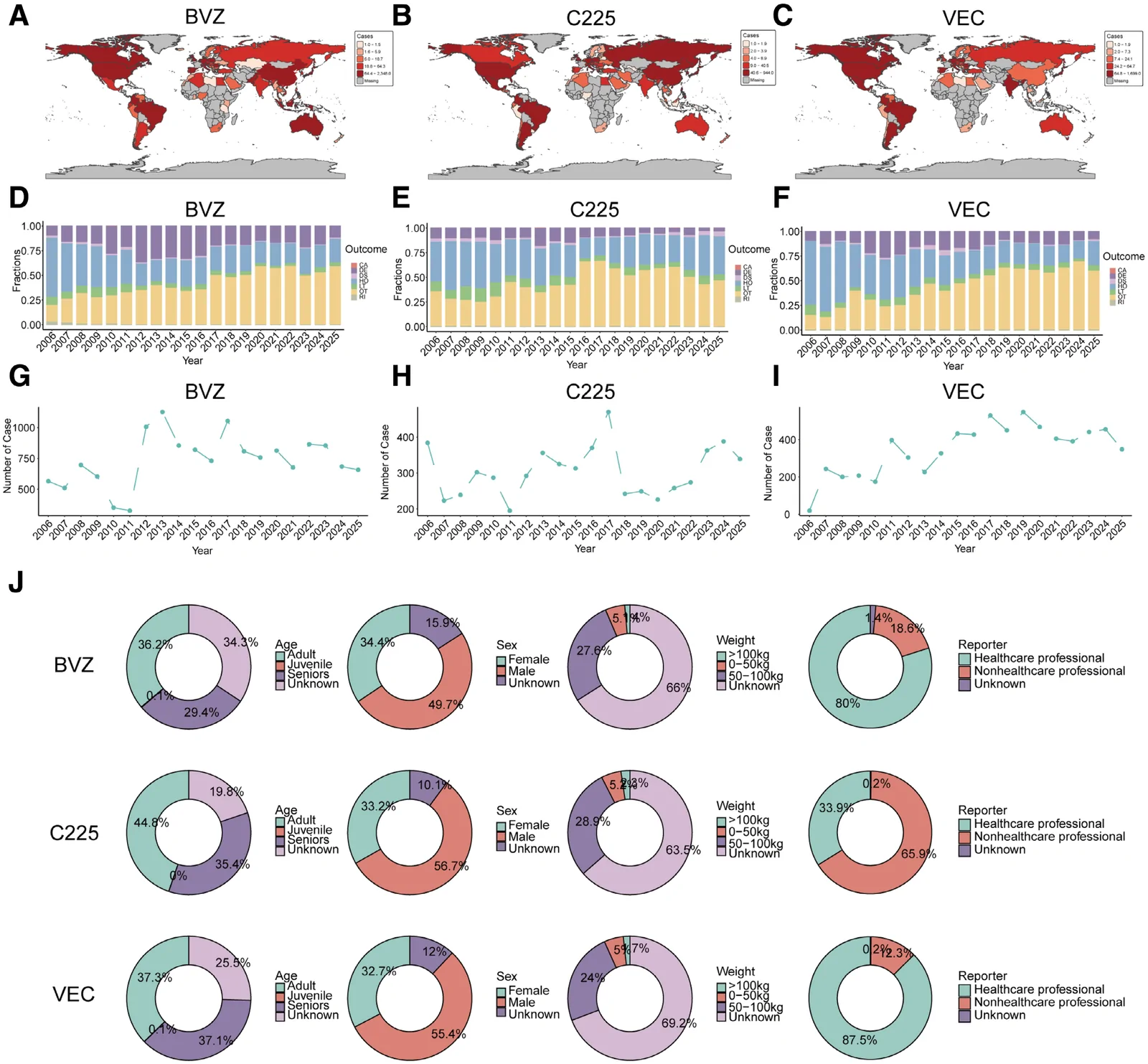
Summary statistics of adverse event reports associated with targeted therapy. Geographic distribution of reporting countries for BVZ (A), C225 (B), and VEC (C); color intensity reflects the number of reports. Annual relative proportions of serious outcome categories for BVZ (D), C225 (E), and VEC (F). Temporal trends in the number of reported adverse events for BVZ (G), C225 (H), and VEC (I). (J) Donut charts summarizing the distribution of patient age, sex, body weight, and reporter type. BVZ = bevacizumab, C225 = cetuximab, VEC = panitumumab.

### 3.2. Spectrum of AEs associated with targeted therapies

Among all reports related to BVZ, the most frequently reported SOC was general disorders and administration site conditions (n = 5840), followed by gastrointestinal disorders (n = 4582), blood and lymphatic system disorders (n = 2123), and infections and infestations (n = 1922). In contrast, disproportionality was highest for blood and lymphatic system disorders (ROR = 4.15, 95% CI 3.96–4.34), with additional signals for skin and subcutaneous tissue disorders (ROR = 2.59, 95% CI 2.44–2.75), hepatobiliary disorders (ROR = 2.84, 95% CI 2.65–3.04), and gastrointestinal disorders (ROR = 2.46, 95% CI 2.37–2.55; Fig. 3A; Table S1, Supplemental Digital Content 1). At the PT level, DE was the most commonly reported event (n = 1705), followed by diarrhea (n = 1265) and disease progression (n = 1250). However, when events were ranked by disproportionality, a different pattern emerged: proteinuria showed the strongest signal (ROR = 27.94, 95% CI 25.08–31.12), followed by palmar–plantar erythrodysesthesia syndrome (PPES; ROR = 18.35, 95% CI 16.36–20.58) and myelosuppression (ROR = 17.86, 95% CI 16.11–19.80; Fig. 3B; Table S2, Supplemental Digital Content 2). Importantly, the palmar–plantar erythrodysesthesia signal in BVZ-containing reports likely reflects concomitant fluoropyrimidine exposure (capecitabine or 5-FU), rather than an isolated BVZ or anti-VEGF class effect. Because FAERS cannot reliably distinguish the contributions of individual drugs in combination regimens, this signal should be interpreted cautiously.

**Figure 3.**
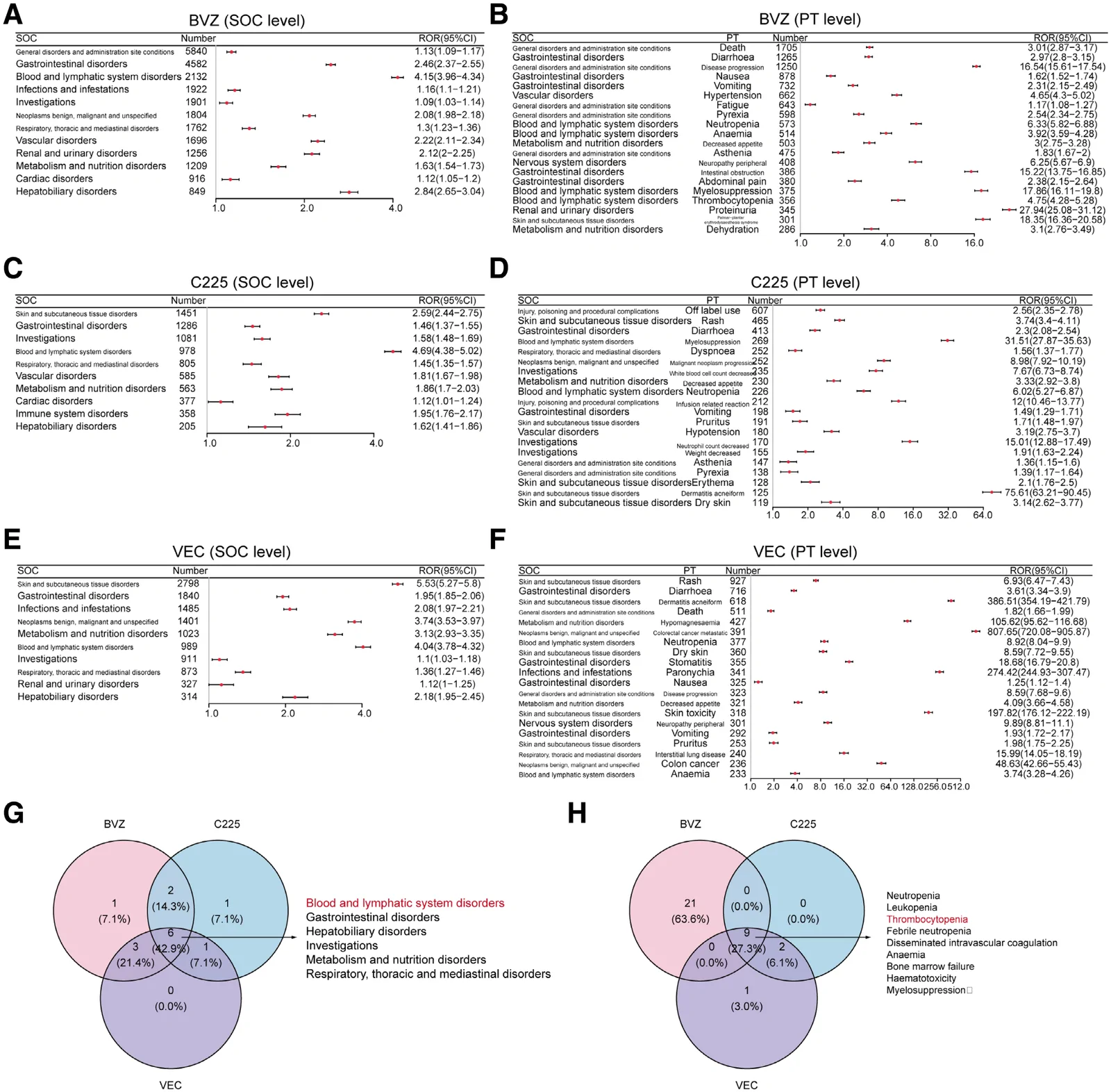
SOC- and PT-level distribution of adverse event reports and disproportionality (ROR) statistics for targeted therapies. SOC-level (A) and PT-level (B) distributions and corresponding ROR estimates for BVZ-associated adverse events. SOC-level (C) and PT-level (D) distributions and corresponding ROR estimates for C225-associated adverse events. SOC-level (E) and PT-level (F) distributions and corresponding ROR estimates for VEC-associated adverse events. (G) SOC categories shared across all 3 targeted agents. (H) PT terms shared across all 3 targeted agents. BVZ = bevacizumab, C225 = cetuximab, CI = confidence interval, PT = preferred term, ROR = reporting odds ratio, SOC = System Organ Class, VEC = panitumumab.

Using the ROR to screen AEs associated with C225, events were most frequently classified under skin and subcutaneous tissue disorders at the SOC level (n = 1451), followed by gastrointestinal disorders (n = 1286), investigations (n = 1081), and blood and lymphatic system disorders (n = 978). When ranked by ROR magnitude, blood and lymphatic system disorders demonstrated the strongest signal (ROR = 4.69, 95% CI 4.38–5.02), followed by skin and subcutaneous tissue disorders (ROR = 2.59, 95% CI 2.44–2.75) and immune system disorders (ROR = 1.95, 95% CI 1.76–2.17; Fig. 3C; Table S3, Supplemental Digital Content 3). For C225, PT-level reporting was dominated by off-label use (n = 607), followed by rash (n = 465) and diarrhea (n = 413). Signal-based ranking again highlighted skin toxicity: dermatitis acneiform was the most prominent association (ROR = 75.61, 95% CI 63.21–90.45), followed by myelosuppression (ROR = 31.51, 95% CI 27.87–35.63) and decreased neutrophil count (ROR = 15.01, 95% CI 12.88–17.49; Fig. 3D; Table S4, Supplemental Digital Content 4).

A similar SOC pattern was observed for VEC, where skin and subcutaneous tissue disorders accounted for the largest share of reports (n = 2798), followed by gastrointestinal disorders (n = 1840), infections and infestations (n = 1485), and neoplasms benign, malignant, and unspecified (n = 1401). Accordingly, skin and subcutaneous tissue disorders (ROR = 5.53, 95% CI 5.27–5.80) exhibited the highest disproportionality, which was followed by blood and lymphatic system disorders (ROR = 4.04, 95% CI 3.78–4.32) and neoplasms benign, malignant, and unspecified (ROR = 3.74, 95% CI 3.53–3.97; Fig. 3E; Table S5, Supplemental Digital Content 5). At the PT level, the most frequently reported events were rash (n = 927), diarrhea (n = 716), and dermatitis acneiform (n = 618). By signal strength, metastatic CRC showed an exceptionally large ROR (807.65, 95% CI 720.08–905.87), followed by dermatitis acneiform (ROR = 386.51, 95% CI 354.19–421.79) and paronychia (ROR = 274.42, 95% CI 244.93–307.47; Fig. 3F; Table S6, Supplemental Digital Content 6).

When results were compared across BVZ, C225, and VEC, 6 SOC categories were consistently shared, including blood and lymphatic system disorders, gastrointestinal disorders, and hepatobiliary disorders (Fig. 3G). At the PT level, 9 events overlapped across all 3 agents, most notably neutropenia, leukopenia, and thrombocytopenia (Fig. 3H). Because thrombocytopenia remains clinically important despite being reported less often than some other events, we selected it as the focus for downstream analyses.

### 3.3. Clinical outcomes and fatality analysis of thrombocytopenia

Across thrombocytopenia reports attributed to BVZ, outcomes were dominated by OT, which accounted for roughly 60% of cases. DE/HO remained relatively uncommon and continued to fluctuate at a low level even after 2015. Starting around 2020, OT began to rise more noticeably (Fig. 4A). For C225, the overall number of thrombocytopenia-related reports was much smaller than for BVZ. OT made up more than 65% of outcomes, while DE was rare (10 cases) and has stayed at a low, flat level over roughly the past decade (Fig. 4B). In contrast, VEC showed a clear surge in thrombocytopenia-related outcomes in 2022 to 2023, with DE + LT + OT all reaching their highest levels – suggesting that the signal has strengthened more recently. Even so, OT remained the predominant category (about 60%; Fig. 4C).

**Figure 4.**
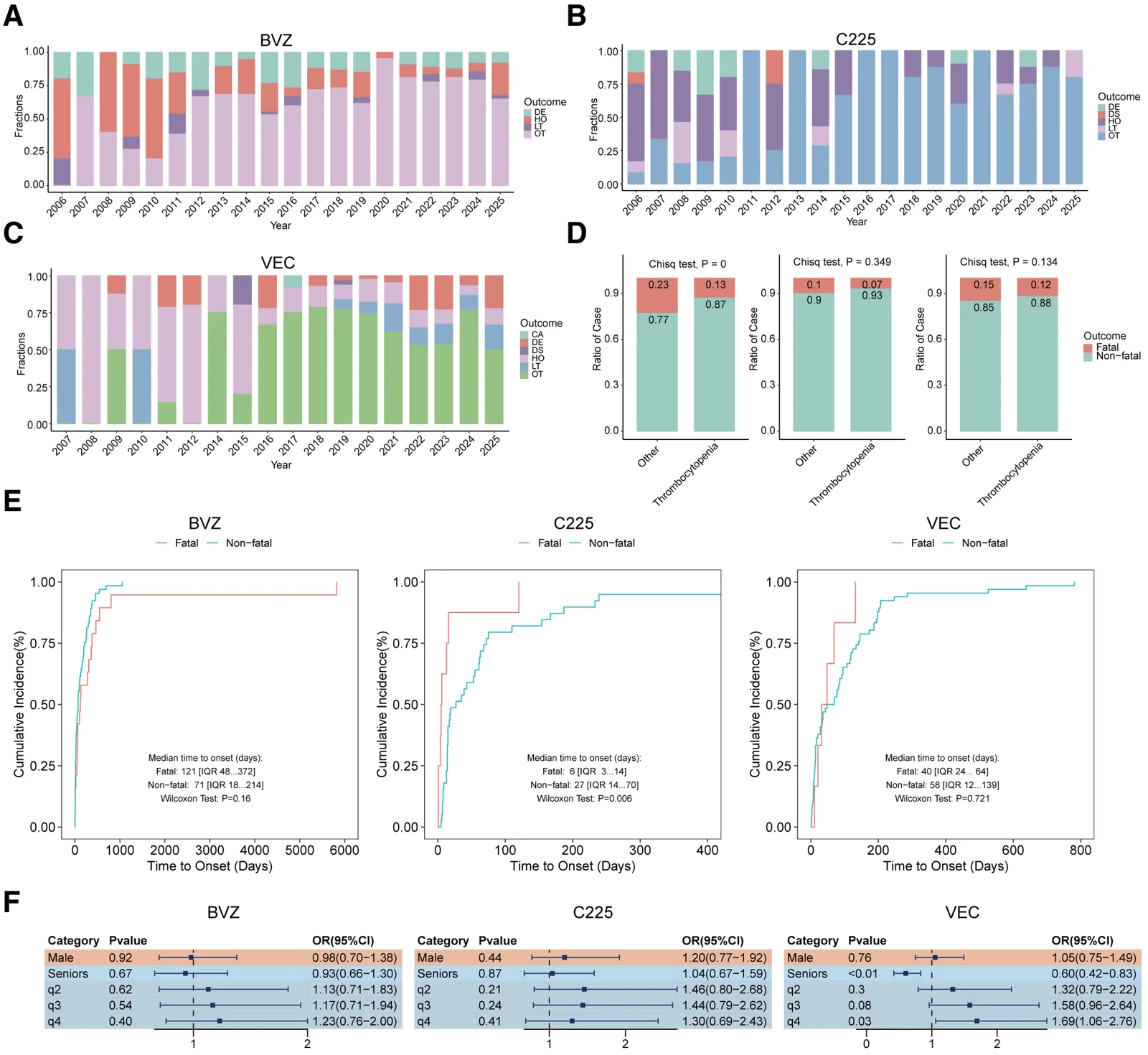
Outcomes, fatality, time-to-onset, and subgroup analyses for thrombocytopenia-related adverse event reports. Distribution of thrombocytopenia-related outcomes for BVZ (A), C225 (B), and VEC (C). (D) Fatality analysis comparing BVZ, C225, and VEC. (E) Time-to-onset analysis for thrombocytopenia-related events associated with BVZ, C225, and VEC. (F) Multivariate logistic regression analysis results for factors associated with thrombocytopenia-related events across BVZ, C225, and VEC. BVZ = bevacizumab, C225 = cetuximab, IQR = interquartile range, SOC = System Organ Class, VEC = panitumumab.

When we compared fatal versus nonfatal outcomes among thrombocytopenia events across the 3 drugs, the fatality proportions were 0.23 (23%) for BVZ, 0.13 (13%) for C225, and 0.10 (10%) for VEC (Fig. 4D). Chi-square testing showed that, for BVZ, thrombocytopenia-related DEs differed significantly from DEs attributed to other AEs (*P* < .001), whereas no significant differences were detected for the other 2 agents. This pattern underscores the need for closer monitoring of bleeding and mortality risk once thrombocytopenia occurs under BVZ, while VEC had the lowest fatality proportion.

### 3.4. TTO of thrombocytopenia

TTO analysis showed median onset times for nonfatal thrombocytopenia of 121 days (IQR 48–372) for BVZ, 6 days (IQR 3–14) for C225, and 40 days (IQR 24–64) for VEC (Fig. 4E). Fatal thrombocytopenia associated with C225 occurred particularly early (median 6 days), significantly preceding nonfatal cases (*P* = .006), underscoring the need for heightened vigilance during the first 1 to 2 weeks of therapy. In contrast, fatal events linked to BVZ occurred later and exhibited broader temporal dispersion, suggesting the importance of prolonged monitoring.

### 3.5. Stratified analysis of thrombocytopenia risk

Given these drug-specific timing differences, we next examined whether patient and reporting characteristics were associated with thrombocytopenia risk using univariable and multivariable logistic regression models. Overall, most covariates showed limited or inconsistent associations across agents. The significant age-related pattern was seen with VEC, where seniors had an approximately 40% lower risk than adults (OR = 0.60, 95% CI 0.42–0.83; *P* < .01). No comparable age effect was evident for BVZ or C225. Seasonal differences were minimal as well. VEC showed higher reporting in Q4 compared with Q1; BVZ and C225 did not show clear seasonality. Finally, sex was not associated with thrombocytopenia risk, and this null finding was consistent across all 3 drugs (Fig. 4F).

### 3.6. Safety signals in combination regimens

Based on regimen-stratified analyses of CRC treatment, BVZ, C225, and VEC combined with FOLFOX or FOLFIRI, clear differences emerged in both the dominant toxicity domains and the specific high-disproportionality signals. At the SOC level, BVZ showed the strongest hematologic toxicity signals under a FOLFIRI backbone, with thrombocytopenia standing out most prominently; hepatobiliary and gastrointestinal disorders also appeared as key reporting domains (Fig. 5A, B). For C225-based combinations, the FOLFOX + C225 regimen showed the strongest signal for thrombocytopenia (ROR = 9.5), underscoring platelet surveillance as a clinical priority. Cutaneous toxicity signals were evident with both C225 + FOLFOX and C225 + FOLFIRI, but tended to be more pronounced in the FOLFIRI setting (Fig. 5C, D). In contrast, VEC was characterized by the most prominent skin and subcutaneous tissue toxicity (ROR ranging from 6 to 6.5). Although its hematologic signal was somewhat weaker than that observed for BVZ and C225, routine hematologic monitoring remains warranted. In addition, the FOLFOX backbone introduced extra signals consistent with neurotoxicity and renal injury (Fig. 5E, F).

**Figure 5.**
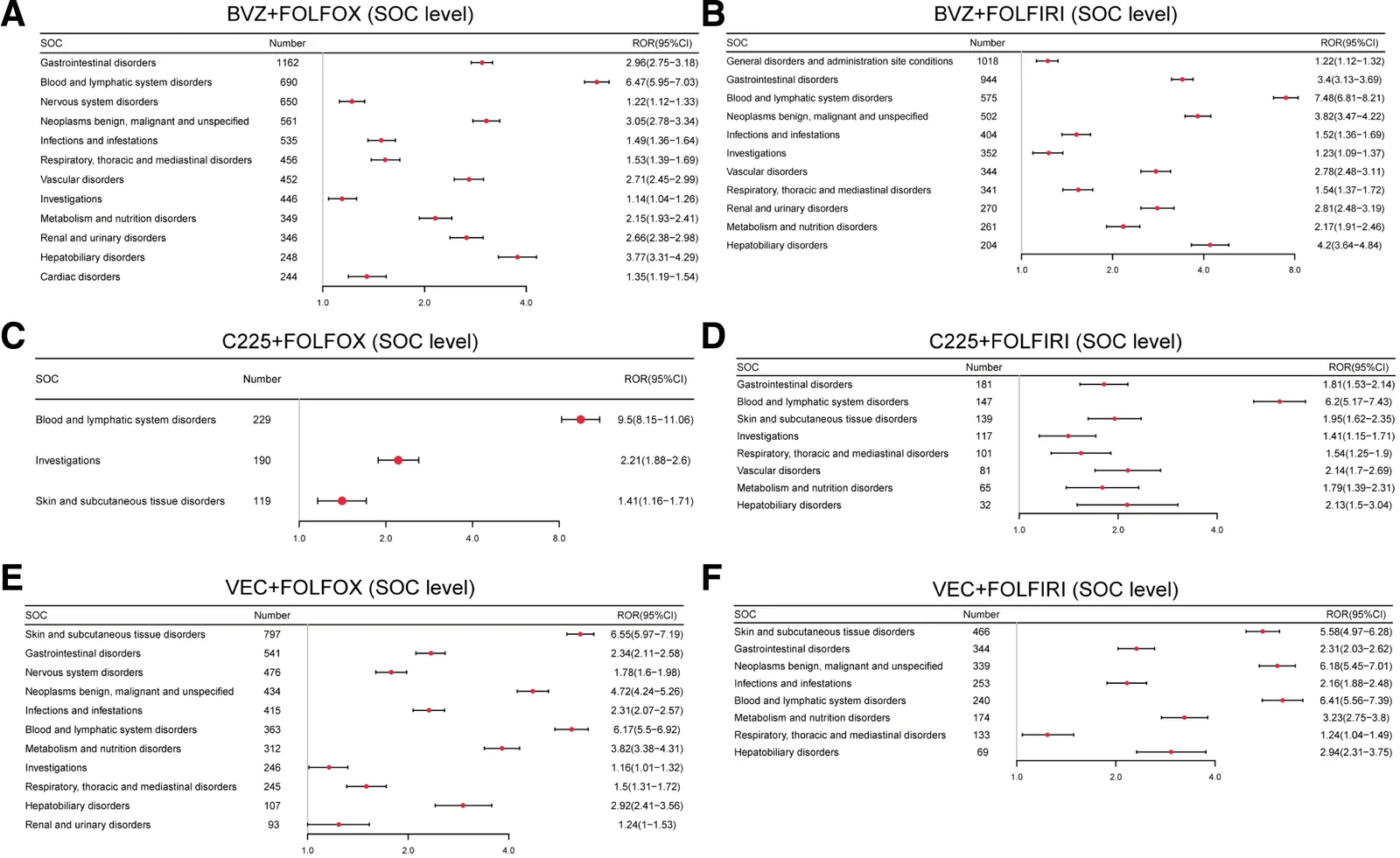
SOC-level adverse event analysis for targeted therapies combined with chemotherapy. SOC-level ROR distributions for adverse events reported with BVZ + FOLFOX (A) and BVZ + FOLFIRI (B). SOC-level ROR distributions for C225 + FOLFOX (C) and C225 + FOLFIRI (D). SOC-level ROR distributions for VEC + FOLFOX (E) and VEC + FOLFIRI (F). BVZ = bevacizumab, C225 = cetuximab, CI = confidence interval, FOLFIRI = folinic acid, fluorouracil, and Irinotecan, FOLFOX = folinic acid, fluorouracil, and oxaliplatin, ROR = reporting odds ratio, SOC = System Organ Class, VEC = panitumumab.

At the PT level, BVZ-containing reports had the largest reporting volume, with prominent signals for proteinuria (ROR range, 37–45) and PPES (ROR range, 27–32; Fig. 6A, B). However, the latter was likely driven by concomitant fluoropyrimidine exposure, particularly capecitabine or 5-FU, rather than an isolated BVZ or anti-VEGF class effect, highlighting potential co-medication bias and confounding by indication. For C225, the strongest signal under FOLFOX was decreased granulocyte count, with an exceptionally high ROR of 476. C225-based regimens also shared signals involving decreased neutrophil count and platelet count, alongside gastrointestinal toxicities such as diarrhea and stomatitis, and a signal for interstitial lung disease, collectively suggesting clinically relevant susceptibility to bone marrow suppression and immune-mediated pulmonary injury (Fig. 6C, D). For VEC, the signal spectrum was dominated by skin-related events, with dermatitis acneiform having the highest disproportionality (ROR ranging from 425 to 533), followed by paronychia (ROR ranging from 276 to 471) and broader skin toxicity signals (ROR ranging from 226 to 355; Fig. 6E, F).

**Figure 6.**
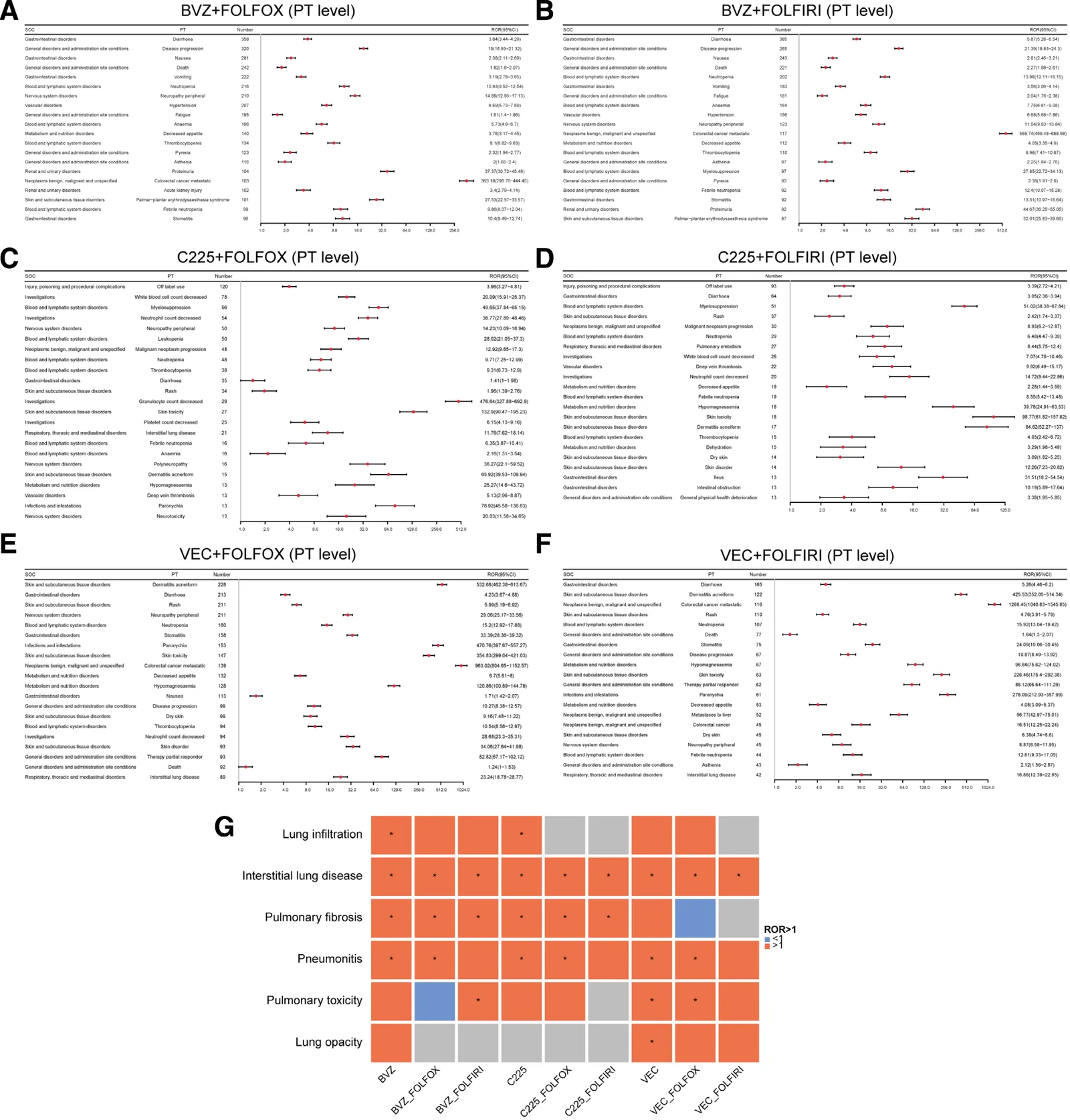
PT-level adverse event analysis for targeted therapies combined with chemotherapy. PT-level ROR distributions for adverse events reported with BVZ + FOLFOX (A) and BVZ + FOLFIRI (B). PT-level ROR distributions for C225 + FOLFOX (C) and C225 + FOLFIRI (D). PT-level ROR distributions for VEC + FOLFOX (E) and VEC + FOLFIRI (F). (G) Heatmap of overlapping adverse events across targeted therapy monotherapy and combination regimens. BVZ = bevacizumab, C225 = cetuximab, CI = confidence interval, FOLFIRI = folinic acid, fluorouracil, and Irinotecan, FOLFOX = folinic acid, fluorouracil, and oxaliplatin, PT = preferred term, ROR = reporting odds ratio, SOC = System Organ Class, VEC = panitumumab.

Notably, peripheral neuropathy emerged as a shared, high-frequency signal only in the FOLFOX setting across all 3 targeted agents (ROR ranging from 14 to 29), consistent with an oxaliplatin-driven overlap toxicity. Taken together, these patterns support regimen-tailored pharmacovigilance priorities: for BVZ, emphasize monitoring for renal/vascular complications and hand–foot syndrome; for C225 + FOLFOX, prioritize prevention and early detection of hematologic toxicity and interstitial lung disease; and for VEC, focus on skin and nail-related AEs. Meanwhile, peripheral neuropathy appears to be a common risk whenever FOLFOX is paired with targeted therapy. Further cross-regimen comparisons showed that a range of pulmonary AEs, including pulmonary infiltrates, interstitial lung disease, pulmonary fibrosis, pneumonia, lung toxicity, and pulmonary opacities – were reported across both targeted monotherapy and combination regimens. This broader distribution argues that pulmonary toxicity may reflect a class-wide vulnerability rather than a risk confined to any single regimen (Fig. 6G).

### 3.7. Thrombocytopenia risk in combination therapy

We next compared the ROR and reporting volume of hematologic toxicity-related PTs across the 3 targeted agents when combined with different chemotherapy backbones. Overall, hematologic toxicity emerged as a shared, class-consistent risk across all targeted regimens, with the strongest signals for granulocytopenia and thrombocytopenia observed in the FOLFOX setting (Fig. 7A). All targeted therapy–chemotherapy combinations yielded positive signals (ROR > 1). The highest disproportionality was seen for VEC + FOLFOX (ROR = 10.5, 95% CI 8.56–12.97), followed by C225 + FOLFOX (ROR = 9.3, 95% CI 6.73–12.9). For BVZ, signal strength was comparable across the 2 backbones (FOLFIRI: 8.98; FOLFOX: 8.10; Fig. 7B). Taken together, these results suggest that FOLFOX substantially amplifies thrombocytopenia risk, and that VEC paired with FOLFOX exhibits the most pronounced disproportionality signal among the evaluated regimens.

**Figure 7.**
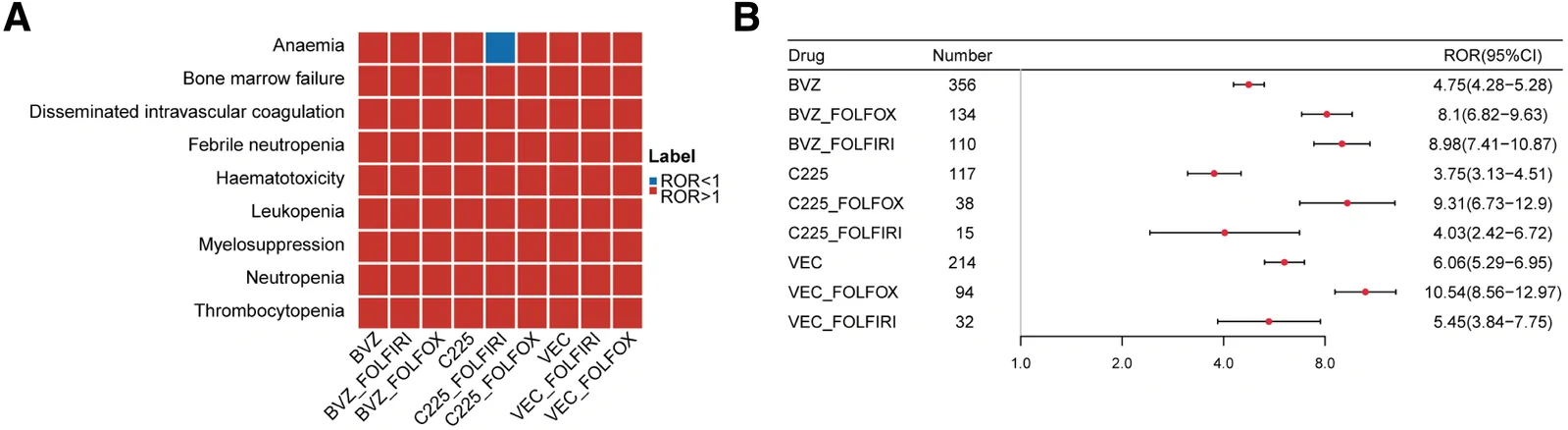
Changes in thrombocytopenia-related risk under combination regimens. (A) Heatmap of ROR estimates for hematologic toxicity-related adverse events. (B) Counts and disproportionality (ROR) forest plot for thrombocytopenia across targeted therapy monotherapy and combination regimens. BVZ = bevacizumab, C225 = cetuximab, CI = confidence interval, FOLFIRI = folinic acid, fluorouracil, and Irinotecan, FOLFOX = folinic acid, fluorouracil, and oxaliplatin, ROR = reporting odds ratio, VEC = panitumumab.

## 4. Discussion

In this study, we leveraged FAERS reports related to CRC targeted therapies recorded from 2006 to 2025 and found that safety signals are not only drug-specific but also strongly shaped by the chemotherapy backbone. BVZ-centered regimens were mainly associated with renal, vascular, and bleeding-related events, particularly proteinuria, whereas C225 and VEC were dominated by dermatologic signals. Importantly, combining these agents with FOLFOX versus FOLFIRI materially altered the AE landscape and its apparent timing, supporting a regimen-based rather than agent-only approach to safety surveillance. Within this framework, thrombocytopenia emerged as a recurrent, clinically consequential event across drugs and combinations, with stronger enrichment in FOLFOX-based combinations, highlighting the need for proactive, regimen-tailored hematologic monitoring.

Our results on BVZ were aligned with established external evidence. The prominence of renal and vascular signals – particularly proteinuria – is consistent with randomized-trial meta-analyses demonstrating increased risks of high-grade proteinuria and hypertension with VEGF inhibition.^[23,24]^ Similarly, the bleeding vulnerability observed across post-marketing datasets is compatible with both meta-analytic evidence and regulatory labeling describing elevated risks of serious hemorrhage.^[25]^ By contrast, the prominent PPES signal identified in the BVZ analysis should not be interpreted as evidence of a direct BVZ-specific toxicity. PPES is a well-recognized toxicity of fluoropyrimidines, particularly capecitabine and, to a lesser extent, 5-fluorouracil.^[26,27]^ Therefore, this signal may reflect residual co-medication confounding, incomplete attribution of concomitant therapy, or reporting of treatment-related events within BVZ-containing therapeutic contexts, even when reports were classified at the agent level. This finding illustrates an important limitation of FAERS: disproportionality analyses can identify drug-event reporting associations but cannot reliably disentangle the contribution of an individual drug from that of concomitant anticancer agents.^[28]^ In our data, thrombocytopenia reported with BVZ was associated with a relatively higher fatality proportion, but this finding should be interpreted cautiously because FAERS outcomes are not adjudicated. In advanced CRC, thrombocytopenia may reflect chemotherapy-related marrow suppression, infection, liver dysfunction, portal hypertension, active bleeding, or their combination, each of which may contribute to HO or DE.^[14,15,25,29]^ Nevertheless, given BVZ’s established hemorrhagic risk, platelet decline during BVZ therapy may warrant careful assessment for anticoagulant use, occult bleeding, and hepatic or portal comorbidity.

For EGFR antibodies, our signal landscape recapitulates the canonical dermatologic dominance of this drug class. Strong signals for acneiform dermatitis, rash, and nail-unit toxicities such as paronychia align with prescribing information and prospective evidence showing that proactive skin-toxicity management can reduce severity and support treatment adherence.^[11,30]^ These findings reinforce that dermatologic AEs are a major driver of supportive-care burden during EGFR-directed therapy and that structured prophylaxis should be considered part of treatment management rather than an ancillary intervention.^[31,32]^ However, several PTs with very high RORs, especially those reflecting metastatic disease or progression, likely represent disease trajectory or coding practices rather than drug-attributable toxicity, highlighting a key limitation of spontaneous reporting systems.^[14–16]^

A major finding of this study is the cross-agent characterization of thrombocytopenia and the demonstration that its prominence and temporal features are strongly influenced by regimen context. In routine oncology practice, thrombocytopenia is often multifactorial, arising from cytotoxic marrow suppression, inflammatory processes, and in oxaliplatin-exposed patients, hepatic sinusoidal injury leading to portal hypertension and splenic sequestration. Oxaliplatin-associated sinusoidal obstruction has been well documented histologically, and spleen enlargement has been proposed as a clinical correlate linked to platelet decline.^[33–35]^ This mechanism is also consistent with our observation that peripheral neuropathy emerged as a shared signal specifically in FOLFOX-based regimens, aligning with the established neurotoxicity of oxaliplatin rather than a primary neuropathic effect of targeted agents.^[36,37]^ Collectively, these findings support a regimen-centered interpretation of toxicity in which the chemotherapy backbone carries dominant organ-system risks while targeted therapies may add distinct toxicity profiles and modify the overall clinical burden of treatment.

The TTO findings further suggest that the timing of toxicity may differ meaningfully across regimens. The early onset of C225-associated thrombocytopenia suggests a need for close hematologic monitoring during the initial treatment cycles, although FAERS data cannot establish whether these early events were directly attributable to C225, the accompanying chemotherapy, infection, or other clinical factors. In contrast, the later median onset observed for BVZ is compatible with cumulative exposure dynamics or downstream complications rather than purely acute hematologic injury, echoing broader observations that VEGF-inhibition toxicities may evolve over longer treatment durations.^[23,24]^ Although FAERS lacks incidence denominators, these temporal differences may help guide surveillance intensity across the treatment course, with early-cycle monitoring for EGFR-based regimens and sustained vigilance when BVZ is continued.

Our study has several strengths. The nearly 2-decade observation window captures changes in prescribing and supportive-care practice, while the CRC-specific, regimen-stratified design improves clinical relevance by avoiding aggregation across heterogeneous tumor types. Combining disproportionality metrics with severity outcomes and TTO provides a more clinically interpretable safety profile than signal detection alone.^[16,29]^ The concordance of major signals with established evidence, including BVZ-related proteinuria and hemorrhage, EGFR-related dermatologic toxicity, and oxaliplatin-associated neuropathy, supports the plausibility of the regimen-linked patterns observed. Several limitations should also be noted. FAERS lacks exposure denominators and therefore cannot estimate incidence or absolute risk. It is also subject to under-reporting, stimulated reporting, incomplete clinical detail, and confounding by concomitant therapy or disease severity.^[14,15,25]^ Importantly, designation of a targeted agent in an individual report does not guarantee isolation from other anticancer exposures, and concomitant or recently administered chemotherapy may be incompletely recorded or incorrectly attributed.^[28,38]^ Consequently, signals such as PPES in BVZ-associated reports may represent toxicity from fluoropyrimidines or other components of the treatment regimen rather than a direct pharmacologic effect of BVZ.^[39]^ Disproportionality metrics are sensitive to reporting dynamics and comparator selection, making them most appropriate for hypothesis generation rather than causal inference.^[16,29]^ TTO fields may be imprecise, and outcome categories reflect reporter judgment rather than adjudicated attribution. These constraints are particularly relevant for rare events and severity comparisons, where modest shifts in reporting can influence signal magnitude. The regimen-specific thrombocytopenia patterns identified here should be prioritized for validation in linked electronic health record or registry cohorts with detailed treatment histories and laboratory trajectories.

## 5. Conclusion

This CRC-focused pharmacovigilance study identified class-consistent and regimen-dependent AE reporting patterns. BVZ was enriched for renal, vascular, and hemorrhagic signals, whereas EGFR antibodies were dominated by dermatologic toxicity. Thrombocytopenia emerged as a shared signal that varied by chemotherapy backbone and was most amplified in FOLFOX-based regimens. These findings emphasize that FAERS safety signals should be interpreted within the context of the complete treatment regimen rather than attributed automatically to an individual targeted agent. They support regimen-tailored monitoring, including early-cycle hematologic surveillance for EGFR-antibody combinations and sustained platelet and bleeding vigilance during BVZ therapy, while providing hypotheses for validation in adjudicated real-world cohorts.

## Author contributions

**Conceptualization:** Hengxue Lin.

**Formal analysis:** Zhihan Li, Gang Li, Hengxue Lin.

**Investigation:** Youqiang Liu, Gang Li.

**Methodology:** Youqiang Liu, Zhihan Li.

**Resources:** Youqiang Liu.

**Writing – original draft:** Youqiang Liu, Zhihan Li, Gang Li, Hengxue Lin.

**Writing – review & editing:** Youqiang Liu, Zhihan Li, Gang Li, Hengxue Lin.












